# Emergency powers, anti‐corruption, and policy failures during the COVID‐19 pandemic in Puerto Rico

**DOI:** 10.1111/lapo.12201

**Published:** 2023-01-26

**Authors:** Jose Atiles

**Affiliations:** ^1^ Department of Sociology University of Illinois at Urbana‐Champaign Urbana Illinois USA

## Abstract

This paper explores how the use of emergency powers by the US and Puerto Rican governments exacerbated the impact of the COVID‐19 pandemic and manufactured the conditions for furthering the multilayered economic, legal, political, and humanitarian crisis affecting Puerto Rico since 2006. The paper discusses three cases. First, it examines how the multiple declarations of the state of emergency, and its constant renewals, produced contradictory public health policies. Since the start of the COVID‐19 pandemic in March 2020, the Puerto Rican government has issued over 90 executive orders aimed at addressing the emergency, producing an unclear, contradictory, and unequal emergency management policy. Second, the paper focuses on the impact of the passing of Law 35 on April 5, 2020, which imposed severe penalties on those who disobeyed executive orders. As a result, hundreds of Puerto Ricans were arrested, fined, and incarcerated for violating the issued order. Third, the paper studies how, citing the presence of corruption, the Puerto Rican government implemented anti‐corruption and anti‐fraud policies that made it more difficult for those most in need of it—mainly poor and racialized individuals, as well as immigrants and working women—to access Pandemic Unemployment Assistance. Thus, the paper argues that emergency policies designed to address the pandemic, punitive governance, and anti‐corruption and anti‐fraud policies undermined Puerto Rico's capacity to handle the pandemic, exacerbated its impact, and created an unequal recovery scenario.

## 1 INTRODUCTION

On February 9, 2021, the bill *Law for Supervision and Accountability in Times of Emergency* (P. del. C. 0515) was introduced in the Puerto Rican legislature. After its approval by the House of Representatives and the Senate on December 1, 2021, it was vetoed by the Puerto Rican governor, Pedro Pierluisi, on January 6, 2022. The bill sought to establish terms and controls on governmental declarations of the state of emergency in Puerto Rico (henceforth PR).^1^ Among other things, the bill aimed to limit the emergency powers of the executive branch, restrict the length of states of emergency, regulate the processes of hiring and buying services during states of emergency, and combat corruption during states of emergency. That is, the bill aimed to create a legal framework for regulating the uses of emergency powers and for creating a clear policy on how to employ such powers during a crisis.

Governor Pierluisi justified his veto by arguing that the PR Constitution, the Political Code of PR,^2^ Law 20 of April 10, 2017,^3^ and US jurisprudence on public health crises^4^ already provided a legal framework for the declaration of a state of emergency, therefore, rendering the bill unnecessary. Furthermore, Pierluisi stated that “this Bill leads us to ask if it is possible to legislate an emergency, and if the very powers that the PR Constitution defines as necessary to deal with emergencies, can be subordinated to the political‐partisan forces that control the Legislature.”^5^ In his veto, Pierluisi further argued that “this Bill is detrimental to the needs of the people in times of emergency,” and “in times when the bureaucracy should not interfere in the mitigation, recovery, and reconstruction efforts that emergencies require. We cannot predict the future, and not all emergencies are the same, so the need to be able to respond quickly is essential.”^6^

The debates surrounding the bill occurred at a time when PR, along with other Global North and South countries, had been actively implementing several legal measures and emergency powers to address the public health crisis caused by the COVID‐19 pandemic (Goitein, 2021; Kipfer & Mohamud, 2021). Some of these interventions included stay‐at‐home orders, lockdowns, quarantines, mask and vaccination mandates, travel restrictions, rules for business operations, restrictions on alcohol sales, and curfews (Burris, Anderson, & Wagenaar, 2021; Burris, De Guia, et al., 2021). The continuous use of emergency measures to contain the impact of the COVID‐19 pandemic and the lack of a well‐defined emergency policy has further eroded Puerto Rican internal democracy and exacerbated previously existing forms of social harm, inequality, and vulnerability. The overuse of emergency powers is particularly evident in how the administrations of Wanda Vazquez (August 2019 to December 2020)^7^ and Pedro Pierluisi (January 2021–present) have managed the COVID‐19 pandemic. For example, between March 12 and December 30, former Governor Vázquez issued 48 executive orders to address the COVID‐19 pandemic,^8^ while Governor Pierluisi has issued 45 executive orders to this same end after taking office on January 2, 2021. As this paper demonstrates, these executive orders and emergency declarations are often contradictory, unclear, and ineffective in addressing the pandemic.

Nevertheless, the use of emergency powers by the PR government is not limited to the pandemic; indeed, PR has been living under a permanent state of emergency for the past 16 years (Atiles, 2020, 2021). To be sure, PR has been experiencing a multilayered economic, legal, political, environmental, and humanitarian crisis, mainly resulting from the fiscal and debt crisis and the structural adjustment programs introduced by the US and the PR governments since 2006 (Garriga‐López, 2020; LeBrón, 2019). It is precisely in this context of multilayered crisis and colonial history that the state of emergency, executive orders, and the emphasis on individual responsibility have become key emergency measures for dealing with any crisis in PR. In the Puerto Rican colonial imaginary, states of emergency and executive orders have become the primary legal and public policy methods for dealing with any crisis affecting the archipelago. The normalization of the use of emergency powers—which this paper conceptualizes as colonial emergency powers—to address every crisis in PR has led to the creation of a policy framework that enables social harm, punitive governance,^9^ and corruption.

By analyzing the case of PR, this paper aims to make a twofold contribution to the law, policy, and emergency powers scholarship. The first is to shed light on the normalization of the use of states of emergency to administer every aspect of colonial life, from legal design and economic development to the management of economic crises, disasters, and public health crises. The second contribution is to show how the use of states of emergency has created a legal framework characterized by punitive governance, corruption, and social harm.

To do so, the article is structured into three sections. In the first section, the paper introduces the theories and debates concerning the uses of states of emergency to address periods of a public health crisis. This first section addresses how the law and emergency powers enable three interconnected practices: overreaching in the use of executive orders and emergency powers, deployment of punitive governance, and corruption. In the second section, the paper provides a brief overview of PR's recent history and its management of the multilayered crisis affecting the archipelago since 2006.

In the third section, the paper analyzes three cases. The first case deals with how the PR government's multiple declarations and constant renewals of a state of emergency have produced a contradictory and often chaotic set of public health policies. The second case analyzes the impact of the enactment of Law 35 on April 5, 2020, which imposes severe penalties on those who disobey an executive order. As a result, hundreds of Puerto Ricans have been arrested, fined, and even sent to prison for violations of executive orders. The third case focuses on how, under the guise of fraud prevention, the PR government implemented anti‐corruption policies that made it more difficult for those most in need of it—mainly poor and racialized individuals, as well as immigrants and working women—to access Pandemic Unemployment Assistance. The three cases aim to provide a context for reflection on the efficacy of law and emergency measures in dealing with crises.

## 2 LAW, EMERGENCY POWERS, AND POLICY FAILURES IN THE COVID‐19 PANDEMIC

Law, legal institutions, and emergency powers have been at the center of managing the public health crisis generated by the COVID‐19 pandemic. Burris, De Guia, et al. (2021, S72) remind us that “the law is integral to effective emergency preparedness and response. It sets out the powers and the duties of officers and agencies, channels resources to individuals and institutions, and sets limits on arbitrary or discriminatory exercise of authority in times of crisis.” Despite the centrality of law in the management of the pandemic, emergency powers and legality have not been free of debate and contention, especially in Global North liberal democracies where misinformation and polarization have defined the societal reaction to COVID‐19.

The following key approaches have mainly defined these academic debates. First, in US academia, there is an important body of sociolegal and political scholarship analyzing the uses of states of emergency by US governors (Curley & Federman, 2020; Deere, 2021; Kettl, 2020; Weissert et al., 2021) and by the US president (Gostin et al., 2020; Pozen & Scheppele, 2020; Rudalevige & Victoria, 2020).

Moreover, there are important comparative analyses of the uses of emergency powers by governments in the Global North (Bolleyer & Salát, 2021; Debre & Dijkstra, 2021; Greene, 2020; Jagannathan & Rai, 2021). Similarly, courts have been at the center of scholarly and political debates during the pandemic (Biehl et al., 2021; Mello & Parmet, 2021, 2022; Parmet, 2021). There have been important debates about the role of the US Supreme Court in reshaping the public health crisis jurisprudence established in *Jacobson v Massachusetts* 1905 (Mello & Parmet, 2021, 2022; Parmet, 2021).

Likewise, the literature emerging from public health and law scholarship has systematically underscored the limits, failures, and contradictions in the legal response to the pandemic. Burris, De Guia, et al. (2021, S72) argue that “COVID‐19 has exposed too many empty promises of equal justice under law.” That is, there has been a massive failure of legal implementation, a lack of legal authority, and inconsistent implementation of legal measures and leadership across many US states and cities. Furthermore, “in some states, governors or legislatures have reacted to COVID‐19 with laws that reflect bad or inadequate policies, delaying state action and interfering with better‐advised local measures through preemptive laws and orders” (Burris, De Guia, et al., 2021, S72).

Pozen and Scheppele (2020) describe how the Trump administration systematically underreached or declined to use its presidential powers to address the pandemic. The authors convincingly demonstrate that overreach or the expansion of emergency powers and executive orders does not have to be the norm. Instead, neoliberal and authoritarian governments can choose not to act or not to use their emergency powers.

Similarly, the discussion of the statutory framework proposed by Rudalevige and Victoria (2020) is illuminating for the analysis of the PR case. In their review, Rudalevige and Victoria (2020) provide an important discussion of the powers and limits of the US statutory framework, including the National Emergency Powers Act,^10^ the Stafford Act,^11^ the Defense Production Act,^12^ the Public Health Service Act,^13^ and several other statutes that enable the use of emergency powers in the United States. This overview is important for analyzing pandemic emergency powers since both US and PR public health policies mainly operate under the protection of these statutes and administrative law. However, unlike the US states and the federal government, in PR this statutory framework operates within the sociolegal context of a colonial relationship. That is, given that PR is an unincorporated territory or colony, the US Congress must, in every instance, determine the extent and scope of applicability of these statutes to PR.

In tandem with this, important debates have emerged in the context of continental political philosophy, particularly in the wake of a series of opinion pieces written by Giorgio Agamben (2021). As a result, several scholars have argued that the management of the COVID‐19 global pandemic has been heavily based on the uses of states of exception, executive orders, militaristic rhetoric, the private sector, and corporations (Agamben, 2021; Appadurai, 2020; Maitra, 2020; Whyte, 2021). A state of emergency is understood as the suspension of the rule of law and civil rights to deal with situations that exceed the “normal” function of the rule of law, such as political violence, disasters, economic crises, and public health crises (Agamben, 2005). Agamben (1998, 2005) argues that the uses of executive orders and invocations of states of emergency to manage these emergencies have normalized the constitution of spaces of *indistinction* or *anomie* between the legal and extra‐legal. As Agamben (2005) suggests, these emergency measures have been normalized in contemporary liberal democracies, becoming—as the management of the COVID‐19 pandemic demonstrates—the apparatus of administration and governance of public and political life.

In addition, the use of states of emergency to deal with crises such as the COVID‐19 pandemic or economic crises has become the legal practice *par excellence* of the neoliberal era, which in turn has normalized the understanding that during periods of crisis regulations ought to be suspended and their application should be disregarded (Parfitt, 2009; Reynolds, 2017; Whyte, 2010). The neoliberal rationale for this is that crises and emergencies need to be addressed through extra‐legal mechanisms and technocratic measures so that the economy and capital accumulation can continue (Maitra, 2020). Thus, although ostensibly tailored to address the preservation of public health, governmental declarations of a state of emergency are designed to maintain existing capitalist accumulation practices and social control structures. These practices of suspending regulations, administrative law, and compliance policies are clearly evident in the PR executive orders that deregulated the governmental procurement of personal protective equipment (PPE) and COVID‐19 test‐kits.

Despite the importance of these debates, the scholarship on the role of law and emergency powers during periods of crisis does not address the experiences of colonial territories and racialized communities like PR. Likewise, these debates have neglected the relationship between colonialism, states of emergency, corruption, and punitive governance. In what follows, I briefly develop the concept of colonial emergency powers to provide a better account of PR's experiences with the management of the COVID‐19 pandemic.

### 2.1 Colonial emergency powers

Contrary to liberal democracies, the state of emergency or exception has always been the rule in colonial settings (Reynolds, 2017; Svirsky & Bignall, 2012). Colonies, and racialized colonial subjects, are placed in a permanent space of anomie, in which their inclusion within the colonial legal system is only possible through their exclusion (Atiles, 2016). Thus, the colonizer always decides which laws and rights do (or do not) apply to colonial societies (Jiménez, 2020; Whyte, 2010).

As Durantaye (2012) and Morton (2013) have argued, Agamben (1998, 2005), despite the centrality of colonialism in the configuration of the paradigm of the state of exception, has ignored its colonial origin and the history of colonialism. For example, Mbembe (2003) has shown that the state of exception is the constitutive legal dispositive of colonialism and has argued that colonial states have used the state of exception as a dispositive of colonial rule. Similarly, Reynolds (2017, pp. 88–89) argues that “the colonial account of emergency cannot be written without reference to capitalist expansion. The use of emergency measures as a form of class repression evolved in concert with colonial expansion, in the context of the broader, mutually interactive relationship between capitalism and colonialism.” Put simply, in the colonies, the state of exception is the norm.

Following this critical tradition, in my previous analysis of the legal constitution of US colonialism in PR, the management of the multilayered Puerto Rican crisis, and the historical process of criminalization of anticolonial movements, I showed that a colonial state of emergency operates in PR. This colonial state of emergency has a dual dimension. On the one hand, the colonial state of exception has been used by the US government as a technique of colonial domination. These techniques of colonial domination include the legal‐colonial design of PR, economic development, tax policies, and the criminalization of anticolonial movements (Atiles, 2020). This dimension of the state of exception is concomitant with the colonial tradition described above. On the other hand, colonial emergency powers refer to the use of states of emergency by the Puerto Rican government to manage crises and disasters and to criminalize sociopolitical movements. Furthermore, colonial emergency powers have also defined the legal framework that has enabled the creation of a tax‐haven‐like economy, state‐corporate crime, and corruption. That is, PR's government and local elites have resorted to the emergency powers to promote economic development and the survival of the colonial economy in times of neoliberal crisis. Colonial emergency powers and the state of emergency have therefore functioned as the administrative ethos and foundational public policy for PR's emergency management for the past 16 years.

I contend that the management of the COVID‐19 pandemic has made the policy failures of the colonial emergency powers clear. To be sure, in PR, that legal framework is the result of colonialism and/or colonial accommodations within the US empire, while the public health policies have been primarily embedded in neoliberal practices and austerity measures imposed to address PR's economic and financial crisis. The interwoven relationship between colonialism, neoliberal practices, and crisis management in PR is precisely what this paper describes as colonial emergency powers.

Furthermore, the few studies that look at the relationship between corruption and the COVID‐19 pandemic mainly emphasize the role of individuals,^14^ overshadowing the role of organized deviance and grand corruption. These studies on cases of individual corruption overlook the negative impact that laws and policies implemented to contravene the impact of corruption can have on poor communities of color. As this paper demonstrates in the case of PR, anti‐corruption policies and legislation create additional hurdles or bureaucratic burdens that limit access to state services and programs such as the PUA.

In his study of the US occupation of Iraq, Whyte (2007) has shown two important aspects of the relationship between emergency powers and corruption. First, he shows how executive orders and the state of emergency created a liminal space for plunder, dispossession, and wealth extraction. Second, he shows that contrary to mainstream analysis, corruption is not the result of exogenous practices, but rather intrinsically embedded in neoliberal rationality and key to capital accumulation. Whyte (2015) further expands his analysis of the political economy of neoliberal corruption, arguing that “various forms of corruption are used to maintain the strategically dominant position of a particular elite group. In other words, corruption in those contexts can be understood as a form of power‐mongering: a means of maintaining economic and political dominance.” (Whyte, 2015, p. 11) In this sense, Whyte's (2015) understanding of corruption emphasizes grand corruption and de‐emphasizes individualistic corruption (petty corruption).   Following this conceptualization, this paper shows how corruption and social harm were a vital part of the management of the COVID‐19 pandemic in PR. The paper argues that the use of anticorruption and fraud prevention policies and legislation limited access for underserved communities to necessary state and governmental resources, exacerbating the impact of the pandemic and the economic crisis it generated.

Finally, following LeBrón (2019), I describe PR's anti‐corruption and anti‐fraud legislation and the policies it implemented to ensure that citizens abided by executive orders as punitive governance. LeBrón (2019, p. 6) states that “punitive governance functions as a form of crisis management that masks an inability or unwillingness to radically transform social relations and institutions in order to address pressing societal problems.” As a crisis management technique, punitive governance is intertwined with emergency powers. Punitive governance has left an indelible mark on how life and death are understood and experienced in PR and has done so in a way that reinforces societal inequalities along the lines of race, class, spatial location, gender, sexuality, and citizenship status. This paper argues that the PR government's management of the pandemic constitutes a particularly exemplary case of punitive governance. As LeBrón (2019, p. 3) points out, “punitive governance functions through an unequal distribution of resources and life changes that affects those populations occupying some of the most tenuous positions in Puerto Rican society.” Indeed, these dynamics are clearly portrayed in PR's management of the COVID‐19 pandemic.

In short, the use of colonial emergency powers entails the normalization of a series of legal, extra‐legal, extractive, and social control practices. It involves the normalization of the state of exception as the mechanism through which colonial life is governed and through which crises in colonial societies are managed. As an emergency management technique, the use of colonial emergency powers is embedded in neoliberal rationality, which implies an unequal distribution of the impact of the crisis. Thus, as I show below, this implies that while the local populations carry the burden of public debt, structural adjustments, austerity, and other crisis management policies, local elites and corporations maintain an administrative structure that enables dispossession and wealth extraction. As a result of this extractive logic, grand corruption, or the corruption of the powerful, is considered a normal or even essential part of the management of emergencies. Nevertheless, these extractive administrative practices weaken the legitimacy of the welfare state on which elites and transnational corporations rely for their accumulation of capital and power. In the face of the progressive erosion of the state's legitimacy, powerful elites deploy punitive governance as a mechanism to exercise social control during periods of crisis. Thus, since colonialism entails a permanent state of emergency/exception, punitive governance has become the key technology through which the state and its citizens interact. Altogether, the concept of colonial emergency powers allows us to better conceptualize the processes and practices that have led to the policy failures in the management of the COVID‐19 pandemic in PR.

## 3 MULTI‐LAYERED CRISIS IN PR: A SHORT HISTORICAL OVERVIEW

Puerto Rico is a Caribbean archipelago consisting of the *Isla Grande*, Vieques, Culebra, and a series of smaller islands. As a result of the Spanish‐American War, the US invaded PR on July 25, 1898, and after the signing of the Treaty of Paris^15^ between Spain and the US, PR was “transferred” to the US. Later, PR became a domain of the US Congress under the Territorial Clause of the US Constitution.^16^ In 1900, the US Congress passed the Foraker Act,^17^ which established a civil government in PR and granted Puerto Ricans limited representation in local government. In 1917, the Jones Act^18^ was passed, granting US citizenship to Puerto Ricans. Simultaneously, between 1899 to 1922, in a series of cases called the *Insular Cases*, the US Supreme Court ruled on what would become the legal definition of PR and the US legal and political relationship with its territories. The colonial status of PR took a new turn in 1952 with the creation of the Commonwealth of PR (under Public Law 600 of 1950 of the US Congress)^19^ and the drafting of a state constitution. This new local government recognized a certain degree of internal democracy and established a republican system of governance within the legal and political framework of US colonialism. This law, however, did not imply a substantial change in the US–PR political relationship; for example, almost all areas related to trade, money, international agreements, immigration, and tariffs remain under US control.

The 1980s marked a period of economic stagnation that culminated in a transformation of PR's economic model and political‐legal discourses. This transition materialized in the 1990s with the transformation of the Puerto Rican economy into a predominantly postindustrial economy based on consumption, tourism, and speculative finance. PR has been experiencing an economic and financial crisis since 2006, resulting from the 1996 US Congress' decision to eliminate the tax exemption law (Section 936 of the US Internal Revenue Code 1976),^20^ PR's high level of public indebtedness, and the local impact of the global economic crisis of 2008. Section 936, which sought to encourage US corporations to establish themselves in PR with the promise of repatriating profits as soon as they were made, was particularly directed at the banking, financial services, and pharmaceutical industries.

The presence of the pharmaceutical industry in PR is relevant for this paper given that two of the major COVID‐19 test‐kit producers,^21^ Abbott and Roche, and the producers of the vaccine, Pfizer, Johnson and Johnson, and AstraZeneca, have been operating in the archipelago under numerous tax exemptions, even after the elimination of Section 936, since the mid‐20th century. For example, these pharmaceutical corporations benefit from Law 135 of 1998 and Law 73 of 2008, which reduce their maximum income tax rate to 4% and grant tax credits for job creation and the purchase of manufactured products in PR. In 2017, the fiscal cost of the tax exemptions provided under these two laws was estimated at around $15.7 billion (Dennis, 2020b). This is more than double the government's operating budget for a fiscal year. These pharmaceutical corporations are also subject to Law 154 of 2010, which imposes a 4% tax on foreign corporations; however, the Federal Treasury reimburses them with a tax credit (Dennis, 2020b). Altogether, these tax benefits created an extractive administrative apparatus in which pharmaceutical and other multinational corporations extract resources and take advantage of the economic policies implemented by the local government but do not distribute the resulting benefits to the local population.

While these corporations have received receive major tax exemptions,^22^ Puerto Ricans have been enduring austerity measures for the last two decades, specifically (1) budgetary cuts, privatization and externalization of public services, low corporate taxation, and high dependence on bonds and debt issuance; (2) the use of emergency laws to deal with the economic crisis and with every other aspect of political life^23^; and (3) the exacerbation of the state‐corporate criminality endemic to colonial systems. These measures led to the bankruptcy of the PR government in 2016, when the public debt totaled $72 billion, and the per capita debt burden was $15,637.^24^

After the default in 2016, the US Congress passed Public Law No. 114–118, known as the Puerto Rico Oversight, Management, and Economic Stability Act (PROMESA). PROMESA represents the US's attempt to address PR's crisis. It is accompanied by the imposition of a seven‐member Financial Oversight and Management Board (FOMB), which oversees the PR government's budget and funds. The members of the FOMB operate as emergency managers in charge of ensuring the Puerto Rican financial system's survival, guaranteeing debt payments, and getting PR back into the financial and stock markets.

Against this background, in September 2017, Hurricanes Irma and María practically destroyed the archipelago. In addition, PR has faced long delays in receiving promised US federal aid for rebuilding infrastructure damaged by hurricanes; numerous corruption cases (Aronoff, 2017; Klein, 2018); and the negligent management of recovery and relief efforts after Hurricane María, which generated close to 4645 deaths (Kishore et al., 2018). The corruption cases continue to affect PR's recovery, to the point that the US government has stopped the transfer of recovery funds due to the PR government's lack of accountability and compliance (Rodriguez, 2020). The negligent management of hurricane María, the multiple corruption cases, and the leak of a now‐infamous Telegram chat (Sosa & Valentín, 2019) generated an anti‐corruption mobilization that resulted in the resignation of Governor Ricardo Rosselló on July 24, 2019.

Wanda Vázquez, former Secretary of Justice, became governor in August 2019 following Ricardo Rosselló's resignation. As previous governors had done before her, on December 31, 2019, Vázquez issued a declaration of a state of emergency to deal with the economic crisis (OE‐2019‐66). This declaration was the fifth extension since the start of the Puerto Rico Financial Emergency. Following this, the Fiscal Responsibility Act (Law 5 of January 29, 2017) was passed by the local legislature.^25^ Then, on June 30, 2020, amidst the COVID‐19 pandemic, Vázquez's administration issued executive order OE‐2020‐50, which extended the state of emergency for the sixth time, leaving it in effect until December 31, 2020. Later, current Governor Pedro Pierluisi extended the state of fiscal emergency in four instances (OE‐2021‐03, OE‐2021‐52, OE‐2021‐84, and OE‐2022‐37). These executive orders denoted the continuation of austerity measures and the permanent state of emergency imposed since 2006 to deal with the economic crisis.

In January 2020, a series of earthquakes devastated PR's southwestern region (Onis et al., 2020), worsening the island's already precarious economic and sociopolitical situation. Governor Vázquez issued 18 executive orders between January 7 and March 12, 2020, and she declared yet another state of emergency to address the aftermath of the earthquakes.^26^ As a result, when the COVID‐19 global pandemic occurred, PR was in a more precarious position than any other state or territory of the US (Pérez et al., 2022). For example, PR was unprepared to face a $9.7 billion direct economic impact, the loss of approximately $2 billion in tax revenue,^27^ the permanent loss of over 100,000 jobs,^28^ and the loss of over 4757 lives due to the pandemic.^29^ Moreover, in 2018, 43% of residents and 57% of children in PR were living in poverty (Balmaceda, 2020a).

In addition, since the 1990 s, PR has been experiencing a public health crisis driven by austerity policies, a lack of resources allocated for preventive health care, and primary care privatization (Pérez et al., 2022). As Pérez et al. (2022, 305–6) remind us, “PR's political subordination to US federal policy has led to the degradation of the public health care system through measures such as chronic underfunding (e.g., through its exclusion from the provisions of the Affordable Care Act) and through the fragmentation and privatization of local health care systems.” Thus, the impact of COVID‐19 was exacerbated by the fact that PR had been enduring austerity measures and a permanent state of emergency to deal with previous disasters. Under these precarious conditions, Puerto Ricans were asked to be resilient, to withstand disasters (without the protection of the US and PR governments), and to be responsible for themselves, their communities, and the local economy.

## 4 COVID‐19 PANDEMIC, COLONIAL EMERGENCY POWERS, AND POLICY FAILURES

This section analyzes three cases that illustrate the law's centrality in managing the pandemic. It also demonstrates the policy failures of the use of emergency powers and the implications of punitive approaches to managing public health crises. To that end, the section analyzes the PR government's multiple declarations of states of emergency, its use of executive orders, and how the constant renewal of states of emergency and curfews has produced a contradictory and often opaque public health policy. Second, the section describes the impact of Law 35 of April 5, 2020, and demonstrates the sociolegal implications of the imposition of punitive governance to address a public health crisis. Finally, the section studies how, under the guise of anti‐corruption, the PR government implemented policies that made it difficult for those who needed it most to access Pandemic Unemployment Assistance (PUA). The aim is to provide the context needed to reflect on the efficacy of the uses of emergency measures to deal with crises.

### 4.1 Facing the public health crisis: Law, states of emergency, and executive orders

When the COVID‐19 pandemic began, Vázquez's administration immediately declared a state of emergency (OE‐2020‐20, March 12, 2020). Between March 12 and December 31, this declaration of a state of emergency was extended and modified 22 times by the Vazquez administration.^30^ In addition, during this period Governor Vázquez issued 48 executive orders to address different aspects of the pandemic. Similarly, Pedro Pierluisi's administration has issued 45 executive orders since January 2, 2021, and has extended or modified the state of emergency in 24 instances.^31^ During the first days of his administration, Pierluisi issued a series of contradictory and sometimes opposed executive orders, many of which amended previous executive orders or disparaged the public health measures implemented by Vazquez's administration. These unclear public health policies constitute an example of policy failure. That is, by producing contradictory and ambiguous information, these executive orders generated the opposite of what the administration initially intended, increasing COVID‐19 cases, misleading people, and creating the conditions for public corruption.

These executive orders used Law 20 of April 2017 to declare a state of emergency.^32^ Law 20 was passed on April 10, 2017, with the intention of creating the Puerto Rico Department of Public Safety (which consolidated PR's various security and public safety agencies). In Article 5.10, the law grants extraordinary power to the governor in case of an emergency. In Section 5.10(b), the law establishes that the governor has the power to amend and revoke regulations and amend and rescind orders as the governor deems convenient during a state of emergency or disaster. It also stipulates that regulations issued during a state of emergency or disaster have the force of law for as long as the emergency or disaster lasts. Furthermore, Sections 5.10(e) and (f) confer power to the governor to acquire goods in times of crisis without the required due process, which is exactly what happened during the early stages of the pandemic when the Vazquez administration acquired personal protective equipment (PPE) and test‐kits, generating several corruption cases (Valentín & Cintrón, 2020). All of these led the legislature to introduce the Bill discussed in the introduction of this article.

As the PR administration has issued emergency measures, Puerto Ricans have been facing exponential increases in unemployment; lack of access to testing and proper monitoring of people infected by COVID‐19, and, during the early stages of the vaccine rollout, limited access to vaccines (Martinez, 2020; Serrano, 2020; Valentín & Minet, 2020)^33^; a dramatic increase in the number of families facing food insecurity (Balmaceda, 2020b); and unequal access to federal emergency relief (Gelardi, 2020). For example, a considerable portion of Puerto Ricans did not receive the economic support provided by the CARES Act^34^ or any other federal aid relief. The different measures taken by the Vázquez and Pierluisi administrations to deal with COVID‐19 have reproduced practices of punitive governance but have also entailed the systemic erosion of an already degraded internal democracy and the exacerbation of an authoritarian style of governing that could be described as autocratic legalism (Scheppele, 2018). A short overview of some of the executive orders issued by these two administrations can shed light on the normalization of the state of emergency and the subsequent policy failures.

For example, executive orders OE‐2020‐22, OE‐2020‐28, OE‐2020‐81, and OE‐2020‐83 activated the PR National Guard (PRNG), allowed the Department of Health to use medical resources from the PRNG, and further militarized public safety. OE‐2020‐23 of March 15, 2020, established a lockdown for 14 days and a curfew from 9 PM to 5 AM, which was amended in several instances until May 2021, when it was finally completely derogated by OE‐2021‐36. Executive order OE‐2020‐24 deregulates procurement processes and the purchase of essential materials such as test kits and PPE.

At the same time, OE‐2020‐27 deregulates the process of hiring experts to manage the crisis. There is a pattern in the strategies implemented by the administration to deal with periods of emergency, consisting of (1) militarization, (2) deregulation, and (3) opening the gates for “external experts” to ply their trade in PR.

Executive order OE‐2020‐29 of March 30, 2020, expanded the lockdown for another 14 days, changed the curfew to 7 PM to 5 AM, and imposed additional limitations on “outdoor activities,” including limiting the circulation of cars on designated days based on their license plate numbers. More restrictions came with OE‐2020‐32, which enforced the mandatory closure of essential services for the weekend of April 10–12. These measures generated a hectic situation in local grocery stores and put Puerto Ricans in a dire humanitarian crisis, to the point that Vázquez's administration had to retract the emergency measures.

While the administration developed contradictory policies, the PR legislature enacted Act 43 of 2020 on April 16, 2020, which provided that COVID‐19‐related medical treatments would be free of charge for the duration of the state of emergency. This measure is similar to the public health measures implemented by the Trump administration, and, while important, it does not address the key limitations of a public health system that has been completely privatized (Pérez et al., 2022)

Furthermore, Vázquez's administration decided to grant immunity from civil cases to “medical facilities and professionals” providing services to COVID‐19 patients through executive order OE‐2020‐36. This provision generated discomfort among Puerto Ricans since it does not guarantee access to PPE or hazard pay for healthcare workers but instead focuses on guaranteeing immunity to corporations that administer private hospitals (Wiscovitch & Sosa, 2020). Similarly, OE‐2020‐40 of May 15, 2020, delineated the process to distribute the $2 billion in relief funds provided under the CARES act. The strategic plan not only set up an unequal distribution of funds (giving more funds to private entities, including hospitals, than to public hospitals and public corporations [Dennis, 2020a]) but also created a new Committee for the Oversight of Disbursements. The committee, made up of members of Vázquez's administration, was asked to establish new accountability and oversight processes, ignoring previously existing emergency relief agencies such as COR3 and further delaying the distribution of funds. This committee remains in place as of April 3, 2022, and it has been further empowered by the Pierluisi administration (OE‐2021‐34), which granted the committee additional authority to administer, allocate, and distribute COVID‐19 relief funds.

Ignoring the recommendations of its own Medical Task Force and pressured by the private sector,^35^ Vázquez issued executive orders OE‐2020‐37 and OE‐2020‐38, establishing the process “to reopen the economy.” OE‐2020‐41 of May 21, 2020, provided more guidelines for “reopening the economy” while maintaining a curfew (7 PM to 5 AM). OE‐2020‐44 of June 12 amended the curfew to 10 PM to 5 AM and paved the way for an almost complete reopening of the economy. Simultaneously with the reopening of the economy, the curfew was extended by every executive order until May 2021, when it was finally eliminated. One of the reasons for not renewing the curfew after May 2021 was the intense opposition to this emergency policy by Puerto Rican civil and human rights organizations (Comision de Derecho Civiles, 2020; Kilometro Cero, 2020).

The reopening of the economy coincided with PR's lack of implementation of expansive testing and contact‐tracing mechanisms, just as the local government had not provided needed information on reported cases (Dennis, 2020b; Sosa & Wiscovitch, 2020). PR lacked the urgent and robust public health capacity needed to care for patients and frontline workers. Thus, the reopening policy backfired in July, forcing the government to walk the policy back (OE‐2020‐54), given the accelerated escalation of COVID‐19 infections and new cases. Nevertheless, the government decided on September 11 to completely reopen the economy and the country while maintaining the 10 PM–5 AM curfew (see OE‐2020‐66). Later on, new restrictions had to be put in place. In the following months, the curfew was amended several times. For example, executive order OE‐2020‐76 eliminated the curfew on October 1, 2020. On October 16, 2020, OE‐2020‐77 reimposed a 10 PM–5 AM curfew, which continued with some variations until its elimination by the Pierluisi administration in May 2021.

Pedro Pierluisi, who took office on January 2, 2021, continued to follow the policies and legal measures developed by the Vazquez administration. For example, on January 2, Pierluisi issued OE‐2021‐01, which mandated that the Secretary of Health develop the necessary mechanisms to administer COVID‐19 tests. Similarly, on July 1, Pierluisi delegated more authority to the Secretary of Health to implement additional prevention policies (OE‐2021‐54). An important difference between the Vazquez and Pierluisi administrations, identified in this paper's analysis of the government's executive orders and the uses of emergency powers, is that Pierluisi has delegated the power to manage the pandemic to executive branch members. The delegation of power was particularly clear in the policies implemented to require travelers to show proof of vaccination or a negative COVID‐19 test (OE‐2020‐51, OE‐2021‐28, OE‐2021‐37, OE‐2021‐40, and OE‐2022‐09). Given its colonial condition, PR could not limit or control its air traffic and/or impose limitations on who entered PR, as such policies fall within the federal domain.

As in the United States, the rollout of the vaccines in PR was conducted mainly by private corporations and legally framed through executive orders. For example, executive orders OE‐2021‐58, OE‐2021‐62, OE‐2021‐63, and OE‐2021‐75 established a vaccine mandate for executive branch employees and contractors. OE‐2021‐63 and OE‐2021‐64 established a vaccine mandate for employees or workers in the service sector (barbers, waitresses, cinema and theater workers, and gym employees, among others). Similarly, OE‐2021‐82, OE‐2021‐87, OE‐2022‐03, OE‐2022‐06, and OE‐2022‐10 required the workers mentioned above as well as students, essential workers, and other members of the service industry to receive a booster shot (in this case, a third dose of the vaccine). In addition, OE‐2022‐15 of February 22, 2022, established further guidelines for the vaccination of students ages five and older.

In December 2021 and January 2022, PR saw an increase in COVID‐19 cases due to the Omicron variant. In response, the Pierluisi administration issued a new set of executive orders and emergency declarations. Although in this case the administration did not impose a curfew, the new orders did include restrictions on mass events (OE‐2021‐80, OE‐2020‐85, OE‐2022‐02, and OE‐2022‐07), additional limitations for travelers (OE‐2021‐81 and OE‐2022‐05), booster shot mandates (OE‐2021‐82, OE‐2021‐87, OE‐2022‐03, and OE‐2022‐06), and orders to close bars, restaurants, and other businesses (OE‐2021‐86).

On March 25, 2022, governor Pierluisi issued OE‐2022‐23, introducing limitations and additional controls for emergency contracts issued during the pandemic. This executive order modified OE‐2021‐21 of March 16, 2021, in which the administration declared a state of emergency in PR's public schools with the goal of contracting out services to improve their infrastructure and facilitating the process of reopening.

The uses of states of emergency and executive orders by the Vázquez and Pierluisi administrations shed light on how the law and emergency powers have been at the center of managing the public health crisis. In this sense, the continued use of emergency powers is embedded in specific forms of neoliberal practices (i.e., privatization of management of the pandemic). This has contributed to the sociolegal transformations that PR is experiencing and the normalization of colonial emergency powers as government policy. In addition, the overuse of emergency powers demonstrates how local administrations have enhanced their administrative control and undermined the powers of the other branches of government.

Conversely, the use of colonial emergency powers and the systematic use of executive orders have not provided relief or recovery for the people. Instead, they have facilitated disinformation, contradictory management of the crisis, and several corruption and fraud cases. Thus, through the above analysis of the PR government's use of executive orders and emergency powers, one can identify the government's policy failures with respect to management of the pandemic.

### 4.2 Act 35 of 2020, punitive governance and emergency powers

While the executive branch was issuing orders, the local legislature approved Law 35 of April 5, 2020, an amendment to Law 20 of 2017 (Article 5.14). This new law criminalizes the spreading of “misinformation during periods of crisis” and any breaching of an executive order that enacts a curfew, lockdown, and/or state of emergency. The sanction and/or penalty for breaching an executive order includes up to 6 months in prison or a $5000 fine; for the spreading of misinformation against an executive order declaring a state of emergency, lockdown, or curfew, the law introduces a fine of $10,000. If the misinformation causes physical harm or severe harm to third parties, the violation is considered a felony.

At this point, we have limited information on how many individuals have been fined, arrested, or prosecuted for spreading misinformation. Nevertheless, thanks to a series of legal mobilizations, requests for information,^36^ and a lawsuit filed by Kilometro Cero,^37^ the PR police department made public the documentation on arrests and fines issued during the first year of the pandemic. In total, 1205 individuals were arrested for violations of executive orders, while 3889 received citations or fines (Kilometro Cero, 2020). The numbers produced by the PR police department are somewhat different from those published in newspapers and in previous investigations carried out by Kilometro Cero.^38^

Furthermore, the official numbers do not reveal that the hundreds of individuals arrested, fined, and even sent to prison for curfew violations came largely from the working poor, Afro‐descendants, immigrants, and other minoritized populations (Florido, 2020; Kilometro Cero, 2020). Arrests were often conducted while these individuals were driving or walking to or from their places of work or running errands such as grocery shopping (Kilometro Cero, 2020). All of this took place as citizens were enduring the effects of quarantine without being provided the necessary information, economic resources, and access to essential services.

The arrests under this law are an exemplary case of punitive governance and colonial emergency powers, insofar as law and emergency powers were used to control, discipline, and punish the most vulnerable populations, rather to prevent the contagion and guarantee the wellbeing of the PR population. Punitive governance transforms the state's emphasis from providing essential services to policing and punishing. An additional dimension of penal governance can be found in the implementation of anti‐corruption laws and policies aimed at preventing fraud in the distribution of PUA.

### 4.3 Anti‐corruption policies and pandemic unemployment assistance

In the context of stay‐at‐home mandates and lockdown, the Trump administration enacted a series of policies intended to address the economic contraction resulting from the public health crisis. Among them was the PUA, which was part of the Assistance for American Families and Workers^39^ program. PUA temporarily expanded unemployment insurance eligibility to self‐employed workers, freelancers, independent contractors, and part‐time workers impacted by the COVID‐19 pandemic in 2020. Similarly, unemployment insurance was expanded, granting additional funds to those who lost their livelihoods due to the pandemic. These programs were established by the Coronavirus Aid, Relief, and Economic Security (CARES) Act,^40^ which expanded the states' ability to provide unemployment insurance to many workers affected by COVID‐19, including people who are not ordinarily eligible for unemployment benefits.^41^ In PR, the distribution and administration of PUA was carried out by the PR Department of Labor (PRDL).

From the beginning, there were a series of cases of corruption and fraud involving the PUA and other funds designed to address the economic contraction generated by the pandemic. According to the US Secret Service, nearly $100 billion has been lost from COVID‐19 relief programs set up to help businesses and people who lost their livelihoods due to the pandemic.^42^ In PR, there have been several reports of fraud cases involving the PUA; however, the PR Department of Justice (PRDJ) and the PR Office of the Inspector General have not provided an official account of these cases.

Nevertheless, it is important to consider how the anti‐corruption and anti‐fraud policies and laws have affected the distribution of economic resources among people in need. I have identified three key patterns. First, the policies were technological measures designed to prevent identity theft and fraud. These technological measures limited access to information concerning the processing of PUA claims.^43^ Under the guise of efficiency and preventing fraud, the PR government externalized the technological services used to process PUA applications.^44^ EVERTEC, a local corporation with ties to governor Pierluisi,^45^ was contracted to run the technological services and the PUA website.^46^ All claims needed to be conducted online, despite the digital divide and the general lack of access to electricity and reliable internet service in post–Hurricane María PR. This exacerbated the lack of access to PUA for certain populations, leaving them with inadequate access or sometimes unable to submit their initial claims.

One of the anti‐fraud measures put in place was a technologically driven system that would raise “controversial points”.^47^ These controversial points, or issues with the application for PUA, entailed that applicants could not get the economic help provided by PUA unless they solved or eliminated those issue with their applications in one of the offices of the PRDL. The key problem was that, given the stay‐at‐home orders, the PRDL offices remained closed, and applicants to PUA were unable to address the issues with their applications. This left numerous unemployed individuals unable to receive PUA. Furthermore, there was no assistance for people trying to make their initial claims, causing many to spend hours on the phone waiting for someone to answer their calls.^48^ The issues with the PUA website and the lack of access to PRDL services led to an official inquiry by the PR House of Representatives on May 11, 2020.^49^ The inquiry found negligent implementation of the website and lack of oversight by the PRDL, and it recommended that the PRDJ conduct further investigations. This case illustrates a dual dimension of the administrative structures of the colonial state. First, it shows how colonial emergency powers are implemented in order to deregulate governmental procurement of services, nearly always without adequate oversight or compliance. Second, it shows how the bureaucratic management of the pandemic contributed to curtailing access to necessary economic resources for those most in need of it. This demonstrates how individuals can become caught between emergency powers and anti‐fraud policies, causing the local state to be unable to provide basic services.

Second, there was inadequate information about how many individuals had received PUA. While there is information on initial claims,^50^ the PRDL did not make public how many people received these benefits. According to the House inquiry mentioned above that took place on May 11, 2020, there were 238,934 applications to the state unemployment insurance program and over 90,000 applications to PUA.^51^ Nevertheless, there is no information about how many of those claims were adequately processed or granted. Furthermore, as Encarnanción (2020) points out, many immigrants, especially Dominican women living in PR, were left out of programs such as PUA due to their migratory status and the lack of concrete policies addressing their needs. Encarnanción (2020) reminds us that undocumented immigrants were excluded from the federal CARES Act. However, while several jurisdictions with foreign‐born populations took steps to fill that gap, PR was not one of them.

Third, the local and federal administrations have been engaging in prosecutions of petty corruption cases. Although the cases were well‐founded in many instances, what struck many commentators was that the people charged with fraud were mainly minoritized individuals, while affluent individuals engaging in fraud against PUA were either not charged, or, if they were, tended to receive deferential treatment from the PRDJ. As Leandra (2021) shows, high school students at an elite private school defrauded the PUA while thousands waited to receive their benefits. On March 24, 2022, local newspapers reported that the PRDL referred over 700 cases of fraud to the PRDJ and the FBI, 600 of which were cases of fraud allegedly committed by incarcerated individuals who had managed to submit applications for PUA while they were incarcerated for offenses not related to PUA.^52^

The PR anti‐corruption and anti‐fraud policies were coupled with the US federal government's punitive anti‐fraud policies. For example, on March 1, 2022, the Biden administration implemented a series of policies targeting PUA fraud and other COVID‐19‐related fraud.^53^ These policies most certainly entail renewed efforts to punish petty corruption.

On September 4, 2021, when the PUA program was finally discontinued, thousands of individuals were left without having received the help and economic support offered by the assistance program. Altogether, while this is an example of how punitive governance specifically operates in PR, it is also an example of how anti‐corruption and anti‐fraud policies often end up targeting minoritized populations. Hence, this study demonstrates how power is exercised simultaneously through a combination of emergency rule and anti‐corruption policies, which in this case entailed both the suspension of the public/private divide (through emergency rule) and its direct re‐inscription (through invoking corruption/fraud). This combination means that people might be cast on either side of that divide—as people of collective concern, or as people self‐interested in private enrichment—unpredictably and politically, reinforcing the power of elites. Thus, administrative law and the emergency powers have been implemented in a way that, rather than enabling humane management of the pandemic, has, in fact, enabled a punitive approach to it.

## 5 CONCLUSION

This paper has aimed to elucidate the role of law in three important aspects of managing the pandemic. First, it has shown how the uses of states of emergency and executive orders to manage the COVID‐19 pandemic and its social, health, and economic consequences are embedded in long‐standing legal and crisis management practices that I have called the colonial emergency powers (by which I mean a normalized state of emergency to manage colonial affairs). These legal and emergency practices include the normalization of the suspension of the rule of law, social harm, and police violence. A telling example is how Governors Vázquez and Pierluisi overreached in their uses of executive orders and imposed draconian emergency measures, such as the approval of Law 35 of 2020.

Second, this paper shows the dramatic impact on specific populations of the emergency measures implemented in PR to manage the pandemic, punitive governance, and anti‐corruption. This is what this paper has identified, in the context of PR, as policy failures—that is, instances in which legal and administrative efforts to address periods of crisis exacerbated the impact of the crisis on underserved and vulnerable populations. These negative consequences are embedded in inequalities, long‐lasting power dynamics, oppression, and organized abandonment. As has been well established, COVID‐19, as with many other crises, mainly affects poor people of color, precarious workers, the elderly, migrants, women, queer communities, and the unhoused. However, colonized subjects in particular, too often a forgotten category in this amalgam of oppression, have been enduring wave after wave of disaster.

Third, the PR case shows how the use of anti‐corruption and anti‐fraud policies exacerbated the impact of the pandemic. This analysis shows that anti‐corruption policies harmed minoritized populations or limited their access to basic state or governmental services. Meanwhile, grand corruption cases remain unaddressed, given that they play an important role in the structure of colonial neoliberalism in PR. The structural dynamics of corruption in recovery and relief efforts drastically exacerbate the impact of crises and, as this paper argues, manufacture other crises.

In this sense, it is worth considering the role of law and emergency powers in managing future public health crises. In PR, as this paper demonstrates, emergency powers and emergency legislation generated the opposite effects of what they intended. That is, rather than enabling an effective and humane response to the pandemic, emergency powers undermined PR's capacity to deal with the emergency. Thus, one must ask what the place of law and emergency power in future pandemics should be. I contend that an effective legal response to a crisis is one that democratizes management and that allows communities to assemble policies that address their needs. Instead of legislating and issuing states of emergency to protect democracy and the rule of law (i.e., instead of resorting to punitive governance), an effective legal response to a crisis should apply the rule of law to enable democratic mechanisms and communitarian responses to crises.

## Endnotes

^1^
PR has been a US unincorporated territory or colony since 1898. For a detailed analysis of PR's colonial and sociolegal history, see Atiles (2016).

^2^
PR Political Code of 1902 as amended. See: https://bvirtualogp.pr.gov/ogp/Bvirtual/leyesreferencia/PDF/Códigos/0-1902/0-1902.pdf (accessed July 30, 2022).

^3^
The law was introduced to create the Puerto Rico Department of Public Safety. Article 5.10 of this law broadens the governor's emergency powers.

^4^
For an analysis of this jurisprudence and the effect of the pandemic on it, see the work of Mello and Parmet (2021, 2022).

^5^
My translation from the Spanish. The original is available here: https://sutra.oslpr.org/osl/SUTRA/anejos/137765/PC0515%20(Conferencia)%20(Veto%20Expreso).pdf (accessed July 30, 2022).

^6^
Ibid.

^7^
Wanda Vazquez, former Secretary of Justice (the PR equivalent of the US Attorney General) of the New Progressive Party (NPP), took office on August 7, 2019, after former governor Ricardo Rossello of the NPP was forced to resign after more than two weeks of popular demonstrations (Cotto, 2020; LeBrón, 2021).

^8^
This is an unprecedented use of executive orders to evade legislative oversight even in time of crisis, and constitutes a clear overreach of the executive powers by the local government.

^9^
For a discussion of the concept of punitive governance, see LeBrón (2019).

^10^
50 U.S.C. ch.36 § 1601 et seq. This statute aims to create the framework for a declaration of a state of emergency in the US.

^11^
42 U.S.C. 5121 et seq. This statute enabled the creation of the Federal Emergency Management Agency (FEMA).

^12^
50 U.S.C. §§4501 et seq.

^13^
58 Stat. 682, Chapter 373.

^14^
See Dincer and Gillanders (2021), Farzanegan and Hofmann (2021), Kohler and Wright (2020), and Zhang (2020).

^15^
Treaty in which Spain transferred sovereignty over PR, Guam, and the Philippines to the US.

^16^
US Constitution Article IV‐3, Clause 2.

^17^
Foraker Act of April 12, 1900 (cap. 191, 31 Stat.77).

^18^
Jones Act, ch. 190, 39 Sta. 951 § 2 (1917).

^19^
Public Law 600, 81st Act of Congress, provides for the organization of a constitutional government by the people of Puerto Rico (July 3, 1950, cap. 446, 64 Stat. 314.).

^20^
26 U.S. Code § 936 ‐ Puerto Rico and possession tax credit. The law came to an end on December 31, 2005.

^21^
For a discussion of the role of the pharmaceutical industry in the management of the COVID‐19 pandemic in PR, see Atiles (2021).

^22^
In the context of the COVID‐19 pandemic, the Trump administration, commentators, and some scholars advocated bringing the pharmaceutical industry “back” to PR (Garcia & Beverley, 2021). This misleading proposal implies that the pharmaceutical industry had left the archipelago, when in fact the pharmaceutical industry totaled $47 billion, or 74% of the total exports of PR. In 2018, 36% of the value of all manufactured products came from pharmaceuticals and medical equipment (Dennis, 2020b).

^23^
It is important to note that every governor since 2006 has declared a state of financial emergency and used executive orders to manage the crisis. These declarations of states of fiscal emergency and the enactment of emergency laws have enabled the local government to issue new bonds that have enhanced Puerto Rican public debt and helped to ameliorate the economic crisis.

^24^
This is ten times higher than that of the average US state ($1419) and nearly three times higher than the highest US state ($5491).

^25^
Law 5 made a legislative declaration of the state of fiscal emergency, legitimating the internal state of emergency as the legal framework for dealing with crises and disasters.

^26^
Governor Vázquez and members of her administration were under investigation for irregularities in the distribution of aid after the earthquakes and for criminal mishandling of the disaster.

^27^
See www.bloomberg.com/news/articles/2020-05-14/covid-19-may-cost-puerto-rico-2-billion-in-taxes-board-says (accessed July 30, 2022).

^28^
See www.estudiostecnicos.com/pdf/Update-COVID-3-ENG-May-18.pdf (accessed July 30, 2022).

^29^
See https://news.google.com/covid19/map?hl=en-US&mid=%2Fm%2F05r7t&gl=US&ceid=US%3Aen (accessed July 30, 2022).

^30^
The executive orders are: OE‐2020‐23; OE‐2020‐29; OE‐2020‐32; OE‐2020‐33; OE‐2020‐34; OE‐2020‐38; OE‐2020‐041; OE‐2020‐044; OE‐2020‐48; OE‐2020‐52; OE‐2020; 54; OE‐2020‐60; OE‐2020‐62; OE‐2020‐64; OE‐2020‐66; OE‐2020‐76; OE‐2020‐77; EO‐2020‐79; EO‐2020‐80; EO‐2020‐81; EO‐2020‐83; and EO‐2020‐87.

^31^
For example see: OE‐2021‐10; OE‐2021‐14; OE‐2021‐19; EO‐2021‐26; OE‐2021‐27; OE‐2021‐28; OE‐2021‐32; OE‐2021‐36; OE‐2021‐40; OE‐2021‐43; OE‐2021‐65; OE‐2021‐67; OE‐2021‐70; OE‐2021‐75; OE‐2021‐80; OE‐2021‐81; OE‐2021‐82; OE‐2021‐85; OE‐2021‐86; OE‐2022‐02; OE‐2022‐07; OE‐2022‐11; EO‐2022‐19; and OE‐2022‐23.

^32^
In addition to Law 20 of 2017, the emergency powers jurisprudence in PR relies on Law 76 of 2000; Act 69 of June 23, 1969, known as the Military Code of Puerto Rico; Act 62 of June 23, 1962; Act 81 of March 14, 1912, known as the Department of Health Act; and the US jurisprudence described above.

^33^
However, recent accounts by the New York Times reveal that 89% of the PR population have received at least one dose, and 78% of the population is fully vaccinated. https://www.nytimes.com/interactive/2021/us/puerto-rico-covid-cases.html (accessed July 30, 2022).

^34^
S.3548–116th Congress (2019–2020), the Coronavirus Aid, Relief, and Economic Security Act, or the CARES Act.

^35^
Composed mainly of the PR Manufacturers' Association, the Pharmaceutical Industry Association of PR, and the Private Sector Coalition.

^36^
This request was conducted under Puerto Rican jurisprudence, which establishes the constitutional right to access governmental information. This was determined by *Soto v. Secretary of Justice*, 112 D.P.R. 477 (1982), in which the PR Supreme Court ruled that the right to access public information is a derivative of the right to freedom of expression enshrined in the Bill of Rights of the Constitution of PR. Additionally, Act 122 and Act 141 of 2019 established the public policy of transparency and access to information.

^37^
Kilometro Cero is a civil society organization mobilizing for police accountability in PR. For more information, visit https://www.kilometro0.org (accessed July 30, 2022).

^38^
See, for example, https://static1.squarespace.com/static/5af199815cfd796ad4930e20/t/5f84f95b5179eb1e3d1174c1/1602550110521/SiPaliques1.pdf; Also, https://www.kilometro0.org/blog-desde-cero/2020/3/15/kilmetro-cero-pide-garantas-para-personas-intervenidas-durante-toque-de-quedanbsp (accessed July 30, 2022).

^39^
See https://home.treasury.gov/policy-issues/coronavirus/assistance-for-American-families-and-workers (accessed July 30, 2022).

^40^
Pub.L. 116–136. March 16, 2020.

^41^
See https://www.dol.gov/coronavirus/unemployment-insurance (accessed July 30, 2022).

^42^
See https://www.cbsnews.com/news/covid-19-relief-programs-secret-service-100-billion-stolen/ (accessed July 30, 2022).

^43^
See https://www.telemundopr.com/noticias/puerto-rico/departamento-del-trabajo-reconoce-problemas-con-sistema-para-solicitar-pua/2075652/ (accessed July 30, 2022).

^44^
From the very beginning, the website experienced technological failures that left hundreds unable to make their initial claims. See: https://www.noticel.com/gobierno/ahora/top-stories/20200508/el-colapso-de-la-plataforma-del-desempleo-se-pudo-haber-evitado/ (accessed July 30, 2022).

^45^
According to the database *LittleSis*, during his time at the law firm O'Neill & Borges (January 9, 2017; July 30, 2019), Pedro Pierluisi served as an attorney and lobbyist for Evertec. See: https://littlesis.org/org/350954-Evertec and https://www.documentcloud.org/documents/6239433-Cabildero-AES.html (accessed July 30, 2022).

^46^
See https://www.noticel.com/ahora/top-stories/gobierno/20200508/evertec-no-facturara-por-la-plataforma-de-pua/ (accessed July 30, 2022).

^47^
See https://www.elnuevodia.com/noticias/locales/notas/esto-es-lo-que-debes-hacer-si-tienes-el-punto-controvertible-3f-tras-reclamar-la-ayuda-por-desempleo/ (accessed July 30, 2022).

^48^
See https://www.elnuevodia.com/negocios/economia/notas/departamento-del-trabajo-procesa-reclamaciones-de-pua-de-manera-manual/ (accessed July 30, 2022).

^49^
See R. de la C. 1767 of May 11, 2020.

^50^
For example, all the initial claims can be found here: https://fred.stlouisfed.org/series/PUAICPR (accessed July 30, 2022).

^51^
According to information gathered by the Federal Reserve Bank of Saint Louis, on May 23, 2020, at the height of the pandemic, there were 67,611 initial claims in PR.

^52^
See https://www.elnuevodia.com/noticias/locales/notas/el-departamento-del-trabajo-ha-referido-sobre-700-posibles-casos-de-fraude-al-pua/ (accessed July 30, 2022).

^53^
See https://www.whitehouse.gov/briefing-room/statements-releases/2022/03/01/fact-sheet-president-biden-to-announce-new-steps-to-combat-criminal-fraud-and-identity-theft-in-pandemic-relief-programs/ (accessed July 30, 2022).
